# Protocol for immunofluorescence detection and quantification of phosphorylated SMAD proteins in human blastocysts

**DOI:** 10.1016/j.xpro.2025.103849

**Published:** 2025-05-28

**Authors:** Todd Fallesen, A. Sophie Brumm

**Affiliations:** 1 Crick Advanced Light Microscopy, The Francis Crick Institute, London, UK; 2 Human Embryo and Stem Cell Laboratory, The Francis Crick Institute, NW1 1AT London, UK

**Keywords:** Developmental biology, Microscopy, Cell Differentiation

## Abstract

The transforming growth factor β (TGF-β) signaling superfamily includes NODAL and bone morphogenetic protein (BMP) signaling, which lead to the phosphorylation of different SMAD proteins and regulate key developmental events. Here, we present a protocol for immunofluorescence detection of phosphorylated SMAD proteins combined with other transcription factors in pre-implantation human embryos. We describe steps for segmenting the nuclei in human blastocysts and quantifying their immunofluorescence intensity. This protocol can be adapted to investigate TGF-β superfamily signaling activity in other mammalian embryos or *in vitro* models of their development.

For complete details on the use and execution of this protocol, please refer to Brumm et al.^1^

## Highlights

• Protocol for detection and quantification of TGF-β signaling in human embryos
• Steps for antigen retrieval of phosphorylated SMAD proteins in human blastocysts
• Instructions for nuclear segmentation using Fiji plugin StarDist
• Details on the use of CellProfiler for nuclear tracking through the imaging z stack

## Abstract (editor highlights)

Publisher’s note: Undertaking any experimental protocol requires adherence to local institutional guidelines for laboratory safety and ethics.

## Graphical abstract

**Figure:**
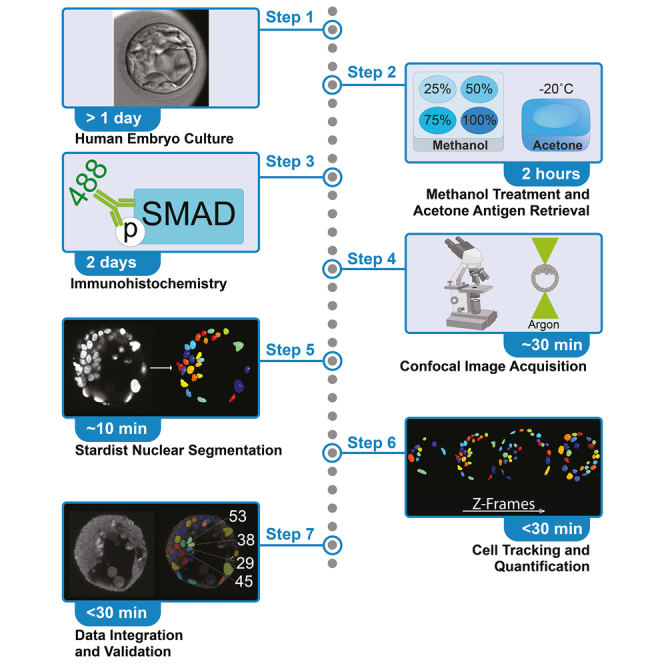

## Before you begin

The transforming growth factor (TGF)-β signaling family is high conserved in deuterostomes,^2^ and is roughly divided into two branches. These branches are represented here by NODAL signaling, which leads to phosphorylation of intracellular effectors SMAD2 and SMAD3,^3^^,^^4^^,^^5^ and bone morphogenetic protein (BMP) signaling, which activates SMAD1/5/9 proteins.^5^^,^^6^

Both NODAL and BMP signaling play key roles in vertebrate embryonic development,^7^^,^^8^ and have been the focus of recent studies on early human development.^1^^,^^9^^,^^10^^,^^11^^,^^12^ Here we provide an immunostaining protocol which enables the detection and subsequent quantification of phosphorylated SMAD proteins in pre-implantation human embryos.

A complete list of required reagents and equipment is provided in the key resources table. Solutions should be prepared fresh. The 4% PFA solution should be no older than 7 days and stored at 4°C.

Aged or inappropriately stored PFA adversely affects the detection of nuclear transcription factors in immunofluorescence analysis of embryos, not just the detection of phosphorylated SMAD proteins specifically. Prepare the solution of Triton in PBS, without calcium and magnesium ions, fresh on the day to ensure optimal washing and permeabilization.

***Note:*** Unless stated otherwise, the manual handling of human embryos was performed using a STRIPPER pipette. Unless stated otherwise, all incubations are performed in 4-well dishes on a rocking platform at standard laboratory room temperature, 15°C to 25°C. The protocol describes the immunostaining of human blastocysts but can also be applied to detect phosphorylated SMAD proteins pre-implantation mouse embryos.^1^^,^^13^ The protocol can be modified to detect phosphorylated SMAD proteins in post-implantation mouse embryos,^1^^,^^13^ with prolonged permeabilization, as well as cultured cells.^1^

### Institutional permissions

This study was approved by the UK Human Fertilisation and Embryology Authority (HFEA): research license numbers R0162, R0397 and R0401 and independently reviewed by the Health Research Authority’s Research Ethics Committee IRAS projects 308099 and 272218. The research was performed in compliance with the HFEA Code of Practice and underwent regular inspections by the HFEA. The human embryos used in this study were surplus and donated by patients undergoing assisted reproduction treatments in the UK. The patients who donated spare embryos were provided with the necessary information about the research project and an opportunity to received counseling. The informed consent included approval of the publication of the results in scientific journals. No financial inducements were offered for donations.

### Preparation of glass capillary

**Timing: 10 min**

1. The glass capillaries for manual handling of human blastocysts are prepared as described in Hosseini *et al.*,^14^ see their Figure 2A1-A5.
  a. Heat a Pasteur glass pipette in a Bunsen burner flame.
  b. Thin by pulling outwards once the glass is malleable.
  c. Break off tip at a size large enough to accommodate an expanded human blastocyst (>300 μm diameter).
    **CRITICAL:** Briefly pass the opening of the capillary through the Bunsen burner flame to soften any sharp edges.
  d. Assess the opening of the capillary under a stereo microscope. Select those with smooth edges, evenly round openings, and sufficient diameter.
    ***Note:*** A suitable pipette can be reused if maintained appropriately: after usage, rinse the glass capillary sequentially with deionized water, 70% ethanol and acetone in this order. To store the capillary safely after use, place an adhesive (e.g. Bostik Blu-Tack) into a suitably large plastic box and stick the capillary on the adhesive to hold it in place. Alternatively, commercially available capillaries may be tried, if the diameter is sufficient.

## Key resources table

**Table:** 

REAGENT or RESOURCE	SOURCE	IDENTIFIER
**Antibodies**		

Rabbit monoclonal antibody anti-phospho-SMAD1/5 (critical reagent) dilution 1:50	CST	Cat# B5B10
Rabbit monoclonal antibody anti-phospho-SMAD2 (critical reagent) dilution 1:50	CST	Cat# 18338; RRID: AB_2798798
Donkey-anti-rabbit, 488 conjugated (critical reagent), dilution 1:300	Thermo Fisher Scientific	Cat# A21206

**Biological samples**		

Vitrified pre-implantation stage human embryos	Bourn Hall Clinic, Bourn, Cambridge, United Kingdom	N/A
Vitrified pre-implantation stage human embryos	Assisted Reproduction and Gynaecology Centre, London, United Kingdom	N/A
Vitrified pre-implantation stage human embryos	The Centre for Reproductive and Genetic Health, London, United Kingdom	N/A

**Chemicals, peptides, and recombinant proteins**		

Global medium (critical reagent)	LifeGlobal	Cat# LGGG-20
HSA protein supplement (critical reagent)	LifeGlobal	Cat# LGP2-605
Mineral oil (critical reagent)	ORIGIO	Cat# ART-4008-5P
Paraformaldehyde (PFA) (critical reagent)	Sigma-Aldrich	Cat# 158127
Phosphate-buffered saline (PBS) with Ca^2+^, MG^2+^ (critical reagent)	Gibco	Cat# 14040-091
Phosphate-buffered saline (PBS) without Ca^2+^ and MG^2+^ (critical reagent)	Gibco	Cat# 15140-122
Triton X-100 (critical reagent)	Sigma-Aldrich	Cat# T8787-100ML
Methanol (critical reagent)	Fisher Scientific	Cat# 67-56-1
Acetone (critical reagent)	Fisher Scientific	Cat# 67-64-1
Solid carbon dioxide “Dry Ice” (critical reagent)	BuyDryIce (optional)	https://buydryice.co.uk/product/5kg-dry-ice-pellets/
Normal donkey serum	Jackson ImmunoResearch	Cat# 017-000-121
DAPI (critical reagent)	Sigma-Aldrich	Cat# D9564
DAPI-containing Vectashield mounting medium	Vector Labs (optional)	Cat# H-1200

**Critical commercial assays**		

BlastThaw kit	ORIGIO (optional)	Cat# 10542010A https://coopersurgical.marketport.net/MarketingZone/MZDirect/Source/cb54879c-c3b4-4b8e-b788-805b80ba9c32

**Software and algorithms**		

CellProfiler 4.2.1 and higher	Stirling et al.^15^	https://cellprofiler.org/previous-releases
Fiji	Schindelin et al.^16^	https://imagej.net/software/FIJI/downloads
Fiji plug-in Stardist	Schmidt et al.^17^	https://github.com/stardist/stardist-imagej
MATLAB	MathWorks	https://matlab.mathworks.com
Anaconda (Python and jupyter)	Anaconda	https://www.anaconda.com/download

**Other**		

Bostik Blu Tack	Bostik (optional)	Cat# 30616595
Bunsen burner head	Fisher Scientific UK (optional)	Cat# 11759317
Bunsen burner cartridge	Fisher Scientific UK (optional)	Cat# 12802154
EmbryoSlide+ culture dish	Vitrolife (optional)	Cat# 16350
EmbryoScope+ time-lapse incubator	Vitrolife (optional)	Cat# 110V-240V 1640
Stereo microscope (critical material)	Olympus (optional)	Cat# sz61-sz51
Stripper pipette	CooperSurgical (optional)	Cat# MXL3-STR
Nunc 4-well dishes	Thermo Fisher Scientific (optional)	Cat# 144444
Embryological watch glass (critical material)	APC pure (optional)	Cat# SDCE4040-1
Glass Pasteur pipette (critical material)	Merck (optional)	Cat# BR747725
Pipette bulb	DWK Life Sciences (optional)	Cat# 292000205
Confocal microscope with argon laser excitation (critical material)	Leica SP8 (optional)	
63× glycerol objective (critical material)	Leica Microsystems	Cat# 11506398

## Materials and equipment

**Table:** 4% PFA

Reagent	Final concentration	Amount
Paraformaldehyde	4%	1 g
PBS with Ca^2+^ and Mg^2+^ Gibco, #14040-091	96%	25 mL
**Total**	**N/A**	**25 mL**

Heat (60°C–70°C) and stir until PFA powder is entirely dissolved (2–3 h). Filter through 0.1 μm sterile filter and store at 4°C for up to 7 days.

**Table:** 0.1% Triton-PBS

Reagent	Final concentration	Amount
Triton X-100	0.1%	10 μL
PBS without Ca^2+^ and Mg^2+^ Gibco, #15140-122	99.9%	10 mL
**Total**	**N/A**	**10 mL**

Vortex until the Triton X-100 is fully dissolved, store at room temperature, 15°C–25°C.

Prepare fresh for each immunofluorescence experiment.

**Table:** 1% Triton-PBS

Reagent	Final concentration	Amount
Triton X-100	1%	100 μL
PBS without Ca^2+^ and Mg^2+^ Gibco, #15140-122	99%	10 mL
**Total**	**N/A**	**10 mL**

Vortex until the Triton X-100 is fully dissolved, store at room temperature, 15°C–25°C.

Prepare fresh for each immunofluorescence experiment.

**Table:** Blocking solution

Reagent	Final concentration	Amount
Normal Donkey Serum (Jackson ImmunoResearch, Cat #017-000-121)	10%	500 μL
0.1% Triton-PBS	90%	4.5 mL
**Total**	**N/A**	**5 mL**

Filter through 0.1 μm sterile filter and store at 4°C for up to 48 h.

Prepare fresh for each immunofluorescence experiment.

**CRITICAL:** Manual handling of human blastocysts requires a stereo microscope. Methanol and acetone are required for antigen retrieval. Watch glasses and a glass capillary are crucial during acetone treatment of human embryos, as acetone dissolves certain plastic ware, which is detrimental to the embryo. It is important that the acetone remains cold while the human embryo is incubating. This especially pertains to the time during transport of the watch glass to and from the freezer. Use watch glasses with a solid glass base, as these maintain a low temperature while handling the embryo under the stereo microscope. If multiple embryos are being handled in parallel, incubate each one in a separate watch glass. Retrieve the embryos individually while the others are kept on dry ice.

***Alternatives:*** The kit required for thawing of human embryos is dependent on the freezing method used by the fertility clinic. To ensure optimal survival of the thawed embryo, the thawing kit corresponding to the freezing method should be used.

Human embryo culture can be performed in alternative incubators which maintain the stated atmospheric conditions.

***Note:*** When using alternative incubators, is it imperative to minimize changes in environmental conditions, such as temperature and gas concentrations. The human embryo should not be removed from the incubator and the incubator should not be accessed unless absolutely necessary. Manual handling of human embryos can be performed with other tools than a STRIPPER pipette. Alternative confocal microscopes and objectives may be used but could impact nuclear segmentation and tracking in the subsequent image analysis pipeline.

## Step-by-step method details

### Culture and fixation of human blastocysts

**Timing: 3.5 h–5 day**s

Here we describe the steps required for culture and fixation of human blastocyst for downstream immunofluorescence analysis.

1. Supplement GLOBAL culture medium with 5 mg/mL protein supplement. Apply 180 μL human embryo culture medium per culture well in an EmbryoSlide+ culture dishes and overlay with 1.6 mL mineral oil.

***Note:*** The mineral oil prevents changes of the media composition due to evaporation, which would adversely affect embryonic development.

2. Equilibrate the EmbryoSlide+ culture dish with human embryo culture medium for at least 8 h at 37°C with 5.5% CO_2_ and normoxia.
3. Thaw an appropriately consented pre-implantation human embryo using the thaw kit matching the freezing method, according to manufacturer’s instructions.

***Note:*** For example Blast thaw kit:

https://coopersurgical.marketport.net/MarketingZone/MZDirect/Source/cb54879c-c3b4-4b8e-b788-805b80ba9c32.

4. Culture the human embryo until the required developmental stage in an EmbryoSlide+ culture dish, using an EmbryoScope time lapse incubator, at 37°C with 5.5% CO_2_ and normoxia.

***Note:*** Depending on the developmental age at which the embryo was frozen, and depending on the required developmental stage for analysis, the culture duration varies. If the embryo was frozen at the desired developmental stage, culture the embryos for at least 2 h post thaw to allow it to recover its morphology prior to fixation.

**Figure 1:**
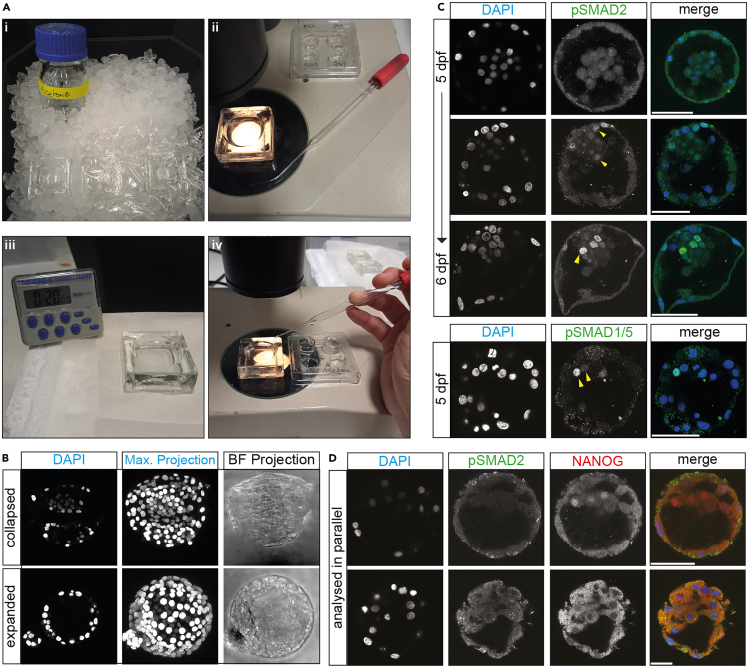
Immunohistochemistry procedure and confocal microscopy data of phospho-SMAD detection in human blastocysts (A) Representative images illustrating key steps in the immunostaining procedure for phospho-SMAD protein detection. (i) Step 5. Acetone and watch glasses for subsequent incubation should be pre-chilled on ice. Covering watch glasses with cling film prevents the build-up of condensate. (ii) Step 11. Transfer of human embryos into pre-chilled acetone using hand-made glass capillaries fitted with a rubber bulb. (iii) Step 11. Once the human embryo has been placed in acetone, the watch glass is transferred onto dry ice immediately. (iv) Step 12. After incubation in acetone, each human embryo is individually recovered into 0.1% Triton-PBS in 4-well dishes. (B) Confocal microscopy detection of nuclear staining (DAPI) in human blastocysts following acetone treatment. Expanded blastocysts can collapse and become flattened (top row). This does not preclude the confocal microscopy data acquisition. (C) Resulting immunohistochemistry detection of phospho-SMAD protein expression in human blastocysts, and nuclear staining (DAPI). No detection of phospho-SMAD2 at 5 days post fertilization (dpf) (top row), emerging phospho-SMAD2 (middle row), and strong phospho-SMAD2 expression (bottom row). Distinct levels of phospho-SMAD1/5 expression are detectable within the same embryo (bottom panel). Yellow arrow heads indicate examples of nuclei expressing the indicated phospho-SMAD protein. (D) Immunohistochemistry of phospho-SMAD2 and NANOG expression in human blastocysts, processed entirely in parallel. Natural variability between human embryos can result in different levels of background staining. Scale bar 50 μm.

5. Pre-chill the acetone and watch glass on ice from the start of this protocol section (Figure 1A-i). Cover the watch glasses with cling film to avoid condensation accumulating as the watch glass cools.

**CRITICAL:** The acetone and watch glasses should be cooled for ∼1 h prior to use.

6. Wash the embryo briefly through 0.1% Triton-PBS.
7. Fix the embryo in 4% PFA in PBS, containing Ca^2+^ and Mg^2+^, for 1 h on ice.

***Note:*** The 4% solution PFA in use should not be older than 7 days. Use PBS with calcium and magnesium ions to maintain the integrity of the extracellular matrix during fixation, which preserves the embryo’s structure.

8. After fixation, quickly wash the embryo in 0.1% Triton-PBS three times to remove excess PFA.

**Potential stopping point:** the protocol can be paused here, to be continued the following day.

However, we do not recommend storage beyond 48 h for detection of phospho-SMAD proteins.

### Immunofluorescence detection of phospho-SMAD proteins in human blastocysts

**Timing: 2 days**

Here we describe the steps required for immunofluorescence detection of phospho-SMAD proteins which allows for detection of additional proteins in human blastocysts.

9. Dehydrate the embryo by sequentially passing it through a dilution series of 25% methanol, 50% methanol, 75% methanol in 0.1% Triton-PBS and undiluted methanol for 5 min each.
10. Rehydrate the embryo through the same dilution series in reverse order for 5 min each.

***Note:*** Prepare two 4-well dishes containing the indicated solutions. Pass the embryo through the sequence of dilution into methanol and re-use the same dishes for rehydration of the embryo.

11. Using a glass capillary, transfer the human embryo into a pre-chilled watch glass containing ice-cold acetone (Figure 1A-ii). Immediately place the watch glass on dry ice to maintain a low temperature and start the 20-min incubation period (Figure 1A-iii).

**CRITICAL:** Acetone dissolves certain plastic ware. The resulting debris can damage the embryo and impair subsequent analysis. To avoid this, use a glass capillary to move human embryos into and out of acetone. Incubate human embryos in individual watch glasses during acetone treatment.

***Note:*** Place a single layer of paper towels on top of the dry ice, to ensure the watch glass is cooled, but dry ice pellets do not adhere to the glass.

12. Using a glass capillary, recover the human embryo from acetone and place into 0.1% Triton-PBS (Figure 1A-iv). Briefly wash the embryo in fresh 0.1% Triton-PBS to remove excess acetone.

***Note:*** Blastocysts can get stuck on the watch glass. Use a P1000 to gently squirt a small amount of room-temperature 0.1% Triton-PBS onto the embryo to dislodge it. Acetone treatment can cause the blastocoel cavity to collapse (Figure 1B). However, this does not preclude downstream analysis.

**CRITICAL:** Warm acetone causes higher immunostaining background. Prolonged incubation in acetone on dry ice is less detrimental than exposing the embryo to warm acetone. If multiple human embryos are being stained, incubate them in separate watch glasses. Retrieve the human embryos from acetone one by one, while maintaining the others on dry ice.

13. Permeabilize the embryo in 1% Triton-PBS, wash three times for 10 min each.
14. Incubate the embryo in blocking solution (10% normal donkey serum in 0.1% Triton-PBS) for 1–2 h at room temperature, 15°C–25°C.
15. Dilute the primary antibody for detection of phosphorylated SMAD proteins at 1:50 in blocking solution. Incubate the embryos in primary antibody solution at 4°C for a minimum of 8 h.

***Note:*** Immunofluorescence detection of other proteins may be affected by this protocol and should be optimized independently.

16. Wash the embryo in 0.1% Triton-PBS three times for 10 min each.
17. Incubate the embryo in secondary antibodies, diluted in blocking solution at 1:300, in the dark for 1–2 h.

**CRITICAL:** We strongly recommend the use of an Alexa 488-conjugated antibody against rabbit IgG to visualize phospho-SMAD protein expression. The 488 fluorophore can be visualized using argon laser excitation, which resulted in stronger detection than white light laser excitation. Strong detection of the fluorophore facilitates detection of low levels of phospho-SMAD protein expression even in a context of high background staining, as is frequently encountered in human embryos.

18. Wash the embryo in 0.1% Triton-PBS with 5 μg/mL DAPI for 5 min, then twice in 0.1% Triton-PBS for 10 min each.

**Potential stopping point:** The protocol can be paused here, to be continued the following day.

19. Mount the embryo in an optical plastic ibidi dish using 250 μL 0.1% Triton-PBS with 1:30 DAPI-containing Vectashield mounting medium per well.
20. Acquire images using an inverted confocal microscope with argon laser excitation and 63× glycerol objective (Figure 1C).

***Note:*** Natural variability between human embryos can result in vastly different result, even if the embryos were cultured, fixed, stained, and imaged in parallel (Figure 1C). In Brumm et al.^1^ the following imaging settings were applied: 1024×1024 pixels, 8-bit pixel depth, using three line-averages and 3 μm z-stack size across the entire sample. The argon laser power was set to 20%, with high laser voltage and detector gain settings to maximize detection of the Alexa488 signal. The downstream image analysis was optimized for the stated imaging parameters. Changing the resolution or Z-stack size may require adjustments in the subsequent CellProfiler pipeline.

**Potential stopping point:** The protocol can be paused here for prolonged time.

### Image analysis and quantification of human embryo microscopy data

Here we describe an image analysis pipeline that will measure the intensity of various nuclear markers in 3D. The pipeline uses StarDist,^17^ a freely available machine learning algorithm to segment nuclei in 2D, and CellProfiler^15^ to measure the intensity of the various markers inside the nuclei, before tracking the 2D nuclei segmentations in 3D space. Matlab or Python is then used to integrate the tracked nuclei to give the total intensity of the markers in 3D.

### Fiji download and code download

**Timing: 30 min**

Here we describe the steps to download the image analysis software FIJI and the code required for the subsequent image analysis.

21. Download the appropriate version of FIJI for your computer at: https://imagej.net/software/FIJI/downloads.

The code used in this section can be found at: https://github.com/todd-fallesen/SHRUMS_Methods.

22. Download into a folder the following three macros:

**Table:** 

Macro name	Function
split_channels_multiple_series_and_rename.ijm	Split a tiff file into separate slices and channels
Stardist_process_folder.ijm	Run StarDist for nuclear segmentation on all nucleus channel images
Enhance_All_C2_C3_c4.ijm	Enhance contrast on images for detection for CellProfiler

**Figure 2:**
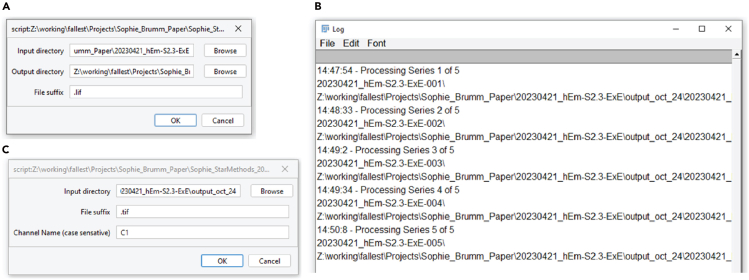
File preparation in FIJI (A) Example of Dialog box for *split_channels_multiple_series_and_rename.ijm*. The script will scan the “Input Directory” and any subdirectories contained therein for any files of the “File suffix” that is given, and split the images into single channel, single slice images. The split images will be saved into a separate folder for each input image. (B) Example of log file of FIJI while *split_channels_multiple_series_and_rename.ijm* is running. This will continuously log while there are images left to be processed by the script. (C) Example of Dialog box for *stardist_process_folder.ijm*. Select as “Input directory” the top level folder containing the output folders from *split_channels_multiple_series_and_rename.ijm.* The script will then proceed through every subfolder and check for any image that ends with the “File Suffix.” Stardist will be run on the Channel specified by “Channel Name.”

23. Install FIJI.
  ***Note:*** FIJI^16^ is a popular fully functional image analysis program which can be downloaded from https://imagej.net/software/FIJI/downloads. In this work, the Linux version of FIJI was used, but there should be no difference between Mac, Windows and Linux versions.
  a. Install FIJI by downloading appropriate version for your operating system. Put the downloaded zip file into a folder in which you want to run FIJI from. Unzip the file in this folder.
  b. Run FIJI.
    ***Note:*** In Windows and Linux, run FIJI by opening the unzipped folder, navigating to FIJI.app, and then running the file ImageJ-win64.exe if Windows or ImageJ-linux64 if Linux. On A Mac, run FIJI by moving the installation file from downloads to applications, right click on it and install as application.
  c. Install the StarDist and CSBDeep plugins needed for this code.
    i. Select Help > Update … on the menu bar. On a new installation, this may take a few moments.
    ii. Click on “Manage Update Sites”.
    iii. Check the box next to “CSBDeep” and “StarDist” in the list of available plugins, then click “Apply and Close”.
    iv. Click “Apply Changes” to install the plugins.
    v. FIJI will prompt for a restart; please restart FIJI.
      **CRITICAL:** On Mac computers, it is not advisable to install FIJI in the downloads folder, as it may not work properly. Move the installation file from downloads to applications, right click on it and install as application.
24. Image processing using FIJI.
  The image processing using FIJI is done in three steps, using three scripts (Figure 2A).
  ***Note:*** To load a script, either drag the script into the FIJI menu bar, or use File→Open and select the script.
  a. *split_channels_multiple_series_and_rename.ijm*: This code is used to prepare the images for use in CellProfiler. Images are split into individual channels and Z-frames using this script. The number of exported images will be equal to the number of channels multiplied by the number of Z-slices in the original image. The script will work on images with multiple series, such as LIF files.
  b. *Stardist_process_folder.ijm*: This code will run a StarDist segmentation on every image of a specified channel. The output of this script is a StarDist label image for each input image.
  c. *Enhance_All_C2_C3_c4.ijm*: See note in step 27.

### Image pre-processing with Fiji

**Timing: 10 min per stack**

Here we describe the steps by which confocal images are processed in FIJI in preparation for subsequent image quantification.

25. Splitting of imaging files into separate channels and Z-frames (Figure 2B).
  a. Load the script *split_channels_multiple_series_and_rename.ijm* into FIJI.
  b. Click “Run” in the bottom left corner of the editor page.
  c. Specify the “Input Directory” where the files to be processed by splitting are located.
  d. Specify the “Output directory” where the resulting split files will be saved.
  e. Specify the file extension or “File Suffix” for the type of image files you have (e.g. .tiff, .lif, .nd2).

***Note:*** The script will process every image in that “Input Directory” that has the specified file extension specified. If the files contain multiple series, such as a .lif file, the script will also process every image in the series. The end result of this script is that images Z-stacks will be separated and labeled by Channel and Z-slice, for input into CellProfiler.

26. Segmentation of nuclei using Stardist in FIJI. (Figure 2C).
  a. Load the script *Stardist_process_folder.ijm* into FIJI.
  b. Click “Run” in the bottom left corner of the editor page.
  c. Specify the “Input Directory” where the files to be processed by StarDist are located. This “Input Directory” is generally the Output Directory from step 25 *split_channels_multiple_series_and_rename.ijm.*
  d. Specify the File suffix (e.g. “.tif”) that the files end with. If the data was preprocessed with *split_channels_multiple_series_and_rename.ijm* the filename will begin with Cx where “x” is the channel number. Specify the “Channel Name (case sensitive)” that has your nuclear imaging.

***Note:*** The script will iterate through every file and subfolder in the “Input Directory” and run StarDist^17^ with its default values on every image from the specified channel and save the output StarDist label images into the same folder as the input images. The filename of the segmentation images will be the same as the input image, but with the word “stardist_” prepended to the filename. The default segmentation model chosen is the “Versatile (fluorescent nuclei)” pre-trained model, which is optimized for nucleus detection. The non-maximum suppression (NMS Postprocessing) parameters default values will generally work quite well on embryo data where nuclei are distinct. If there are many false positives in the detection, increasing the Probability/Score threshold value will decrease the number of false positives, at the expense of fewer segmentations and a greater amount of missed segmentation (false negatives). If there are many overlapping segmentations, the overlap threshold may be increased, but in the authors’ experience, this leads to many false positives.

### Additional image pre-processing

**Timing: 10 min per stack**

Some data may need further pre-processing before use in the CellProfiler pipeline. In the test data for Brumm et al.,^1^ additional pre-processing was done on the imaging data before measurement. This pre-processing is done on the images that have gone through the initial FIJI pre-processing, which separates and labels images by Z-slice and Channel.

***Note:*** Channel 2 (psmad): The image is thresholded, and the threshold is used as a mask on the original data. The masked original data is then median filtered with a radius of 1. The result is an image where background is reduced, and the image is slightly smoothed.

Channels 1, 3, 4: Remove outliers is run on the image, with a radius of 6 and a threshold of 120. This means that if a pixel intensity deviates from the median of the surrounding pixels in a radius of 6 pixels by more than 120, it will be replaced by the median of the surrounding pixels in that radius. A further median smoothing of a pixel radius 2 is then done on the image. This reduces overly bright puncta in the image, which are often biologically irrelevant.

27. Background reduction and contrast enhancement (optional step)
  ***Note:****Image using Enhance_All_C2_C3_c4.ijm* is an optional script to reduce the background noise and enhance contrast of images before processing in CellProfiler. The script will iterate through every file and subfolder in the “Input Directory” and run the enhancement on any files it finds. For any images from channel 2 it will prepend the prefix “Contrast_enhanced_” to the original image filename when saving the enhanced image, for all other channels the script will prepend the prefix “Enhanced_” onto the original image filename while saving the enhanced image file. All enhanced images will be saved into the original image folder.
  a. Load the script into FIJI.
  b. On lines 22–25, comment out using ‘//’ at the beginning of any line, any channel you don’t want to pre-process.
  c. Click “Run” in the bottom left corner of the editor page.
  d. Specify the “Input Directory” where the files to be processed are located. This “Input Directory” is generally the Output Directory from step 25 *split_channels_multiple_series_and_rename.ijm.*

### Quantification of segmented nuclei using CellProfiler

**Timing: <30 min per embryo dataset**

CellProfiler^15^ is a general purpose image analysis tool, useful for developing reproducible and reusable pipelines to quantify data in large image sets (https://cellprofiler.org). CellProfiler is a module based program, where each image analysis task is performed by a distinct module which has inputs, outputs and changeable parameters. Here we will review the 4 channel pipeline. The 5 channel pipeline is equivalent, with an addition of the 5^th^ channel.

***Note:*** CellProfiler Projects are saved as two files, a ∗.ccproj and a ∗.cppipe file. The main difference is that the *cpproj* file has the image dataset information, as well as the paths for saving data saved into it. For headless CellProfiler use (i.e. on a computing cluster) you would use the *cppipe* file. When using the Graphical User Interface (GUI) version of CellProfiler, you can load either file. In CellProfiler, help and explanation on any parameter can be accessed by clicking on the question mark next to that parameter.

**Figure 3:**
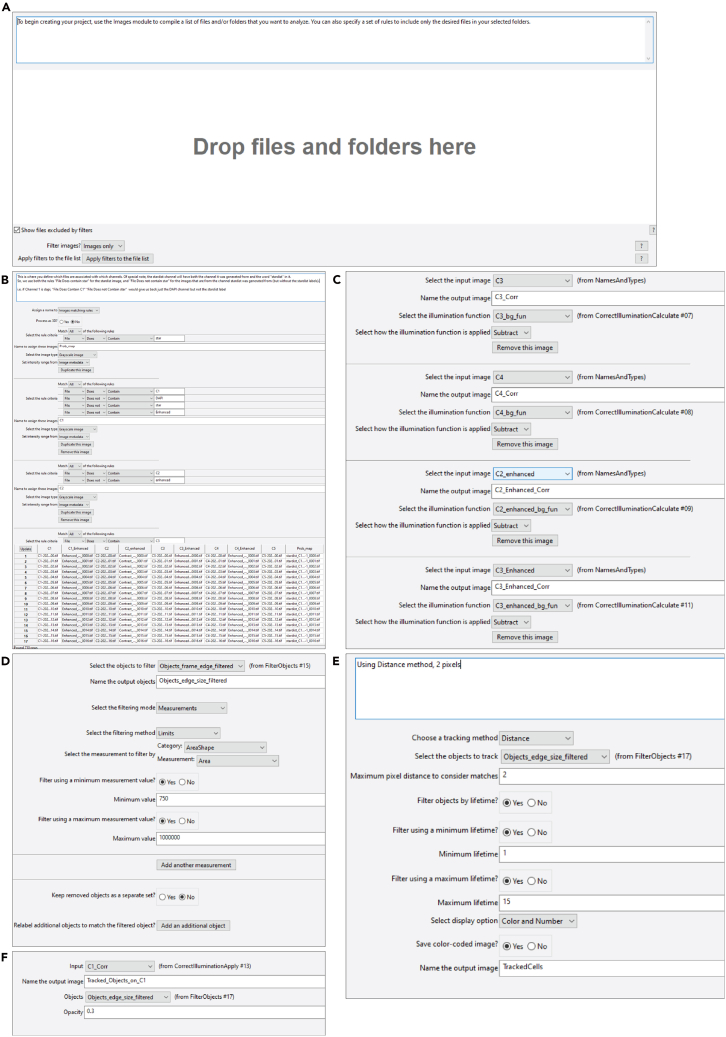
Image analysis in CellProfiler (A) Visualization of the Images module in the CellProfiler pipeline where you drag the images that you wish to process in the CellProfiler pipeline. Individual files, groups of files, or folders can be dragged into this area. (B) Example of specifying criteria in the NamesAndTypes module to differentiate the loaded images into different channels and image sets. (C) Configuration of CorrectIlluminationApply example. It is important to match the input images with the associated background functions. (D) Example of configuration in FilterObjects module to filter by size. In this case, all objects that are less than 750 pixels in area are removed. The maximum value is set at a very large value to ensure that objects aren’t removed for being too large. For objects that are on average smaller or larger, the minimum size value can be changed here. (E) Example of TrackObjects module configuration. In this configuration, objects are tracked from slice to slice using the distance between their centers, with a maximum distance they are allowed to move of 2 pixels. The lifetime has been configured to have a maximum of 15 slices, i.e. the maximum thickness of a nucleus can be 15 slices. (F) Example of configuration of OverlayObjects module, to overlay objects onto an image. In this example, the StarDist segmentations after filtering to remove those under 750 pixels area and those touching the image edge are removed and overlaid over the C1_Corr image. The opacity is how dark the objects will appear on the underlying image. On some systems, this needs to be quite low, ∼5% or 0.05.
  b. In the Metadata module, answer the question “*Extract metadata?* “with *NO.*
    ***Note:*** This pipeline doesn’t use extracted metadata to differentiate image sets.
  c. Under the NamesAndTypes module select “No” to the radio button “Process in 3D?”. This pipeline is not set to process in 3D.
  d. Configure the NamesAndTypes module to differentiate between image sets by filename, using a rule system which uses pattern matching in the filenames to specify to CellProfiler what the images are.
    ***Note:*** The NamesAndTypes module is used to determine which images correspond to which channels, i.e the DAPI, StarDist and bright-field images, as well as the other 3 channels (Figure 3B). For example, to specify the image set that is the StarDist segmentation maps, the first rule that would be specified is “File does contain: star” to find only the images that contain the word “star” in the filename. To access the rules, under the drop-down menu next to “Assign a name to” choose “Images Matching rules”. A single option for a rule is then presented on the screen. To add more rules, click the “+” sign at the end of the line for the rule criteria. To further specify another image set after specifying the rules for one image, choose the option “Add Another image” and specify the rules for that image set. All of the image rules used in this pipeline to specify the images are listed below:
  e. Match all the following rules:
    i. StarDist Channel:
       File does contain star (User sets “star” here)
       Name to assign these images Prob map (user sets Prob map here)
    ii. Channel C1:
       File Does Contain C1.
       File Does not Contain DAPI.
       File Does not Contain star.
       File Does not Contain Enhanced.
    iii. Channel C2:
       File Does Contain C2.
       File Does not Contain enhanced.
    iv. Channel C3:
       File Does Contain C3.
       File Does not Contain Enhanced.
    v. Channel C4:
       File Does Contain C1.
       File Does not Contain Enhanced.
    vi. Channel C2_enhanced:
       File Does Contain C2.
       File Does Contain enhanced.
    vii. Channel C3_Enhanced:
       File Does Contain C3.
       File Does Contain Enhanced.
    viii. Channel C4_Enhanced:
       File Does Contain C4.
       File Does Contain Enhanced.
    ix. Channel C1_Enhanced:
       File Does Contain C1.
       File Does not Contain DAPI.
       File Does not Contain star.
       File Does Contain Enhanced.
  f. Under the **Groups** module, click *No.* There isn’t a need to group the images here.
30. Configure the Background correction modules:
  ***Note:*** This set of CellProfiler modules calculate a background function for the different image sets and perform background correction. The CorrectIlluminationCalculate modules are used to calculate the background illumination function across all images for a given channel which accounts for any inhomogeneity in the illumination.^18^ The illumination functions are used in CorrectIlluminationApply to correct any background inhomogeneity, by subtracting off background from the images (Figure 3C).
  a. Configure the CorrectIlluminationCalculate module.
    ***Note:*** There are 8 modules here, each corresponding to one of the input images (C1-C4, and C1-C4_enhanced). For each CorrectIllumnationCalculate module, there is one input image, and one output image. For example, here we configure the first of the 8 modules for Image C1 the Input Image is C1 (from NamesAndTypes).
    i. Set Output Image to C1_BGfun (Background illumination function). Under the “Select how the illumination function is calculated” dropdown box, choose “Background.”
    ii. Set the block size to an appropriate size for your data set.
      ***Note:*** The block size is found empirically across the entire dataset, and "should be large enough that every square block of pixels is likely to contain some background pixels, where no objects are located" (quoted from https://cellprofiler-manual.s3.amazonaws.com/CellProfiler-4.2.5/modules/imageprocessing.html#correctilluminationcalculate).
      For the provided data, a size of 75 pixels was found to be best.
    iii. Under the “Rescale the illumination function” select “No”.
      ***Note:*** CellProfiler automatically uses pixel values between 0 and 1 normally. Rescaling the image will change pixel values so they are greater to or equal to 1. This is used when correcting an image by dividing the image by a calculated background function. In this pipeline, the background function is subtracted from the image, so rescaling is not recommended.
    iv. Under “Calculate function for each image individually or based on all images” select “All: Across Cycles.”
      ***Note:*** This gives a background image that will be subtracted from all images, based on all images.
    v. Select “Fit Polynomial” under the “Smoothing Method” and do not retain the averaged image or retain the dilated image.
  b. Configure the CorrectIlluminationApply module.
    ***Note:*** This module applies the background correction function calculated in the previous modules to the image of interest.
    i. Under “Select the input image” select from the dropdown menu choose an input image (C1-C4 or C1-C4_enchanced) and then choose the associated background correction function calculated using CorrectIlumminationApply (Figure 3C).
    ii. Enter a name for the output image, and then under “Select how the illumination function is applied” choose “Subtract.”
      ***Note:*** Subtract is chosen as the illumination correction function that was chosen was “Background” and it is recommended to use “Subtract” in this case.
    iii. When the parameters have been filled out for one image, click on “Add another image” underneath the last parameter set in order to add another.
    iv. Under the two radio boxes, “Set output image values less than 0 equal to 0?” and “Set output image values greater than 1 equal to 1?” select “Yes.”
31. Configure the StarDist Segmentation Conversion using ConvertImageToObjects.
  ***Note:*** This module converts the StarDist image of nuclei labels into an object mask. These masks will later be used to quantify the intensity of various channels in the nuclei.
  a. Select “Prob_map” as the input image.
  b. In the text entry box “Name the output object”, name the objects *“ConvertImageToObjects.”*
  c. For the radio box “Convert to boolean image” choose “No” and for “Preserve Original labels” select “yes.”
    ***Note:*** Preserving original labels keeps the same labels as were generated by StarDist in FIJI. A Boolean image will have labels that are only 0 or 1, and thus overlapping segmentations will not be separated.
32. Configure the FilterObjects module.
  a. Select “ConvertImageToObjects” under the dropdown menu for Input objects and specify the “Output objects” as “Objects_frame_edge_filtered.”
  b. Under “Select the filtering mode” choose “Image or Mask Border.” Do not keep removed objects as a separate set.

***Note:*** This filter removes any segmentation masks that are touching the edge of the image. This is done to remove any nuclei that aren't completely imaged.

28. Opening project in the CellProfiler GUI.
  a. Open CellProfiler (version 4.21 or higher)
  b. Open the analysis pipeline by selecting File→Open Project and selecting *SHRUMS_4_Channels_Enhanced_Channels_For_Star_Methods.cpproj.*
29. Configure the File Processing Modules:
  ***Note:*** The Images, Metadata, NamesAndTypes and Groups modules are involved in inputting images and files.
  a. In the Images module, drag and drop the directory with the single channel single plane images into the window marked “*Drop files and folders here.”* (Figure 3A).

### Identification and classification modules

The following section provides details on the modules of the pipeline, which are responsible for measuring the size and intensity of the objects, filtering the objects by size and tracking them in Z for 3D reconstruction.

33. Configure the MeasureObjectSizeShape module.

Select the box for“Objects_frame_edge_filtered” under “Select object sets to measure”.

***Note:*** The objects to be measured are chosen by selecting them with the appropriate checkbox. The measurements in this step are size and shape parameters which will be used to filter out the objects under a certain size in the next step.

34. Configure the second FilterObjects module.
  **CRITICAL:** This filter removes any objects that smaller than 750px in size. This corresponds to an object diameter of ∼30px (Figure 3D). This size was chosen to correspond to the size of the nuclei as they make their first and last appearances in an image stack. By removing the initial and final image planes of the nuclei, the tracking algorithm in the next step is much better able to differentiate nuclei. If there is still quite a bit of tracking object fusion, this variable would be good to vary.
  a. Specify Input objects are “Objects_frame_edge_filtered” in the drop down menu.
  b. Specify Output objects are “Objects_edge_size_filtered.”
  c. Choose “Measurements” under the dropdown menu “Select the filtering mode.”
    ***Note:*** These are the measurements taken in step 33.
  d. In “Select the filtering method” choose “Limits” and then select the Category “AreaShape” and under “Measurement” choose “Area.”
  e. Select “Yes” for both “Filter using a minimum measurement value” and “Filter using a maximum measurement value”, the minimum value is 750 and the maximum value is 1000000.
    ***Note:*** Change the minimum value to change the size of the nuclei excluded. The maximum value was chosen to not exclude any objects for being too large.
35. Configure the TrackObjects module.
  Specify that the “Objects to track” are “Objects_edge_size_filtered” (Figure 3E).
  ***Note:*** The TrackObject module is used to track objects between Z-slices, so that they can be later reconstructed into 3D objects.
  a. Select “Distance” under Choose a tracking method, with the “Maximum pixel distance to consider matches” set to 2.
    ***Note:*** This variable sets how far the object can move from plane to plane and can be varied to tweak tracking.
  b. Choose “Yes” under “Filter objects by lifetime” to give a lifetime of objects, in this case from minimum of 1 to maximum of 15 images, chosen to eliminate objects that persist through the entire image.
  c. For troubleshooting, choose “Yes” under “Save color-coded image”, which will allow you to evaluate the tracking manually. In this pipeline, these images are named by default “TrackedCells.”
    ***Note:*** This module is conventionally used to track objects through time; here it is being used to track objects through space in the Z-direction, allowing us to integrate all the image planes corresponding to single nuclei. The real output of this module will be in the final output spreadsheet, where it will give a Tracked ID Number for each object in each image, so that the integration of the nuclear planes can be done using the Tracked ID Number.
36. Configure the MeasureObjectIntensity module by selecting the checkbox for “Objects_edge_size_filtered” in the “Select objects to measure” box.

Under the “Select images to measure” box, select the checkboxes for C1, C1_Corr, C1_Enhanced, C1_Enhanced_Corr, C2, C2_Corr, C2_Enhanced, C2_Enhanced_Corr, C3, C3_Corr, C3_Enhanced, C3_Enhanced_Corr, C4, C4_Corr, C4_Enhanced, C4_Enhanced_Corr.

***Note:*** This module has no immediate output, but the intensity for each channel selected and will be appended to the information about each object in the final exported data.

37. Configure the MeasureObjectSizeShape module by selecting the checkbox for “Objects_edge_size_filtered.”

***Note:*** Output of this function is size and shape measurements of the objects after filtering and tracking. They can be used for further analysis, such as normalizing by object area.

### Data analysis modules

The following modules of the pipeline are responsible for measuring the size and intensity of the objects, filtering the objects by size and tracking them in Z for 3D reconstruction.

38. Configure the OverlayObjects modules by specifying in each module, the objects dropdown box that are overlaid objects are “Objects_edge_size_filtered”, and specify the Opacity is 0.3 (Figure 3F). Further configure each module to overlay images as in Table 1.

**Table 1:** Relations of input and output objects in the OverlayObjects modules

Module_Number	Input image	Output image
1	C1_Corr	Tracked_Objects_on_C1
2	C2_Enhanced_Corr	Tracked_objects_overlay_C2_Enhanced_Corr
3	C2	Tracked_overlay_on_C2_original
4	C1_Enhanced_Corr	Tracked_Objects_on_C1_Enhanced
5	C3_Enhanced_Corr	Tracked_objects_overlay_C3
6	C4_Enhanced_Corr	Tracked_objects_overlay_C4

**Figure 4:**
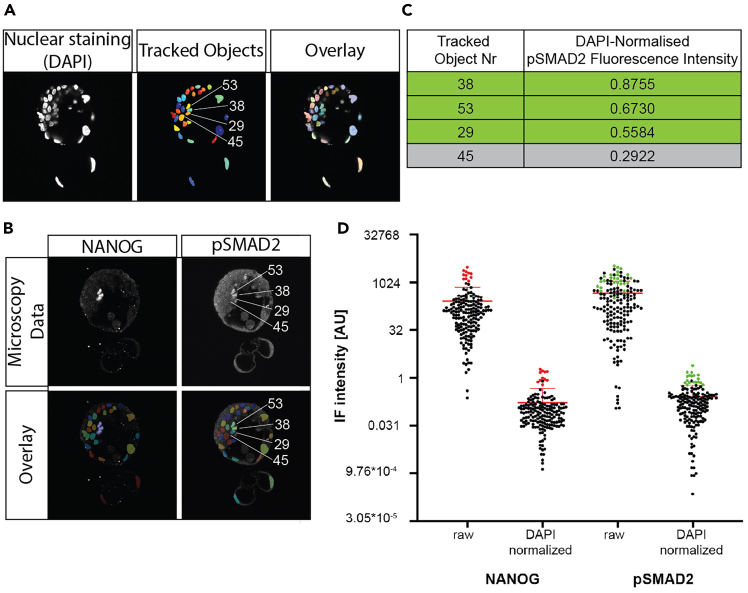
Manual validation of nuclei with high DAPI-normalized fluorescence intensity (A) Nuclear staining (DAPI) in a hatching human blastocyst (left), the resulting tracked objects (middle) and the overlay onto the original microscopy data (right), to illustrate the fidelity of nuclear segmentation and tracking. (B) Immunohistochemistry detection of NANOG and phospho-SMAD2 expression in the same Z-plane displayed in (A) and overlaid with the tracked nuclei (bottom row). (C) DAPI-normalized phospho-SMAD2 immunofluorescence intensity of nuclei highlighted in and, in (A) and (B). Nuclei 38, 53, and 29 (green) had confirmed phospho-SMAD2 expression, while nucleus 45 (gray) does not display phospho-SMAD2 expression. (D) Bee swarm plot of NANOG and phospho-SMAD2 fluorescence intensity of all nuclei in one analyzed human blastocyst, either raw or DAPI-normalized. Error bars indicate mean +/- SEM. Highlighted are nuclei with manually validated expression of NANOG (red) or phospho-SMAD2 (green). DAPI normalization results in a reduction of false-positive phospho-SMAD2-expressing nuclei amongst the tracked object with highest normalized phospho-SMAD2 immunofluorescence intensity.

***Note:*** This module is used to overlay the objects that were filtered and tracked over the original images in order for manual inspection. These steps are done to verify that the pipeline is working properly (Figures 4A and 4B; Table 1).

39. Configure the SaveImages modules.
  ***Note:*** The SaveImages modules save the overlay images and tracking images to the default output folder, as specified in the Output Settings (Table 2). We use consistent naming structures here so that they are easily comparable in other software such as FIJI.

**Table 2:** Overview of Output images from CellProfiler Pipeline created by the SaveImages modules

Module number	Image to save	File prefix	Sub-folder
1	Tracked_Objects_on_C1_	Tracked_objects_on_C1_	C1_overlay
2	Tracked_overlay_on_C2_original	Tracked_objects_overlay_C2_original_	C2_original_overlay
3	Tracked_objects_overlay_C2_Enhanced_Corr	Tracke_objects_c2_enhanced_bg_corr	Tracked_objects_c2_enhanced_bg_corr
4	Tracked_objects_overlay_C3	Tracked_objects_overlay_C3_	C3_overlay
5	Tracked_objects_overlay_C4	Tracked_objects_overlay_C4_	C4_overlay
6	TrackedCells	Tracked_cells_	TrackedCells
7	C2	C2_original_	C2_uncorrected
8	C2_Corr	C2_corrected_	C2_bg_corrected
9	C2_Enhanced_Corr	C2_Enhanced_Corr	C2_Enhanced_Corr
10	C3_Enhanced_Corr	C3_Enhanced_Corr	C3_Enhanced_Corr
11	C4_Enhanced_Corr	C4_Enhanced_Corr	C4_Enhanced_Corr
12	C2_enhanced_bg_fun	C2_bg_function_	C2_bg_function
  For all modules, verify the following parameters are set:
  a. Select type of image to save: “Image”
  b. Select method for constructing file names: Sequential Numbers.
  c. Number of digits: 4.
  d. Saved file format: tiff.
  e. Image bit depth: 8-bit integer.
  f. Save with lossless compression? : No.
  g. Output file location: Default Output Folder sub-folder.
  h. Overwrite existing files without warning?: No.
  i. When to save: Last Cycle.
  j. Record the file path information to the saved image: No.
  k. Choose the appropriate parameters for “Select the image to save”, “Enter file prefix” and “sub-folder” from Table 2.
    ***Note:*** The original channel 2 image is saved, along with the background corrected, and enhanced versions of Channel 2, as well as the background function as well, so that comparisons can be made to see if the illumination correction is being performed correctly.
40. Configure the ExportToSpreadsheet module by specifying the prefix of which is specified in the box “Filename prefix.” There is an option to “Select the measurements to export” to which “yes” has been chosen.

***Note:*** The output will be a set of csv files, and the measurements needed are already selected. They should only be edited with a good understanding of implications, as alterations of this may cause the downstream MATLAB processing to fail.

### Data processing using MATLAB

**Timing: ∼1 min per image set**

Matlab is used to perform data analysis on the output from CellProfiler. The input of the Matlab script is the Output directory from CellProfiler. The CellProfiler output is structured so that each data set (i.e. each embryo) is in a subfolder within the default output directory.

***Note:*** CellProfiler measures the intensity for each 2D StarDist segmentation across all images in the dataset. The 2D StarDist segmentations are tracked in 3D space using the overlap tracking algorithm built into CellProfiler, and the tracking ID is used to integrate the 2D objects and their intensity values into 3D objects. The result is an intensity value for each 3D object for each input channel. We have provided two MATLAB data processing files, SHURMS_Wrangling_4_channel.m and SHRUMS_Wrangling_5_channel.m. These are functionally the same, with just an addition of the 5^th^ channel. Here we continue to demonstrate the use of the 4 channel pipeline.

41. Open SHRUMS_Wrangling_4_channels.m in Matlab to run the file.
42. Configure the essential parameters before running the script:
  a. *input_path*: The path to the default output folder from CellProfiler.
  b. *filename*: The filename of the csv file from CellProfiler that contains the intensities and the tracking data. This is by default:
     'Stardist_ Labels_Enhanced_ImagesObjects_edge_size_filtered.csv'
  c. *save_file_name*: This should be a meaningful name that must end in “.xlsx”.
    ***Note:*** These inputs are specified on lines 31–33 of the Matlab code.
    input_path = '/path/to/default/Output_Folder/'; %the directory where all the output subdirectories are
    filename=’Stardist_Labels_Enhanced_ImagesObjects_edge_size_filtered.csv';%the file we will be looking for in each subdirectory
    save_file_name = 'Date_Meaningful_Filename_All_Channels_Output.xlsx';
    Example of code that needs to be changed to run the Matlab script.
43. Click the green “Run” button to execute the code, once the input_path, filename and save_file_name are set.

***Note:*** The output from the Matlab script is an Excel file for each dataset, and one master Excel file for the entire processing run, with each dataset as a separate tab in the file.

### Data processing using Python (alternative step)

**Timing: ∼1 min per image set**

While the authors used Matlab to perform data analysis on the output from CellProfiler, we have included a Jupyter Notebook written in Python which will do the same analysis as the Matlab script. As in the Matlab Script, the input to the Jupyter Notebook is the Output directory from CellProfiler. The CellProfiler output is structured so that each data set (i.e. each embryo) is in a subfolder within the default output directory.

***Note:*** This was not used in the original analysis, and is provided for ease of accessibility. We have provided two Python data processing files, SHURMS_Wrangling_4_channel.ipynb and SHRUMS_Wrangling_5_channel.ipynb. These are functionally the same, with just an addition of the 5^th^ channel. Here we continue to demonstrate the use of the 4 channel pipeline.

44. Install Python with jupyter notebooks to run the file. If you do not have a Python installation, we recommend using anaconda, if available (https://www.anaconda.org) as a Python manager.

***Note:*** Many institutions have IT rules in place governing the installation of Python on computers, as such for initial installation of Python, it may be best to seek advice from a local IT department.

45. Install numpy and pandas packages to use this notebook.

***Note:*** We recommend using pip to install both numpy and pandas.

46. Open the Jupyter notebook “SHRUMS_Wrangling.ipynb” in Jupyter notebooks.
47. Select a code block and press “shift-enter” on your keyboard to run the code and progress to the next block.

**CRITICAL:** Blocks must be run in order.

***Note:*** The first code to be run imports the numpy and pandas packages for data analysis.

48. Specify the input_path, filename and save_file_name In the second code block.

***Note:*** These are identical to what is done in Matlab (see step 42, and code block below)

input_path = '/path/to/default/Output_Folder/' #the directory where all the output subdirectories are

filename='Stardist_Labels_Enhanced_ImagesObjects_edge_size_filtered.csv' #the file we will be looking for in each subdirectory

save_file_name = 'Date_Meaningful_Filename_All_Channels_Output.xlsx'

#this is the output file name

49. Continue running the code blocks in order to import the data, print out the number of data folders there are, and to run the complete analysis.

**Table 3:** Explanation of the values exported by CellProfiler for each column in the exported .csv file

Column name	Value
Tracked_Object_Number	3D Object number
Intensity_IntegratedIntensity_C5	Total intensity enclosed by 3D Segmentation ROI, integrated over all slices, C5
Intensity_IntegratedIntensity_C1	Total intensity enclosed by 3D Segmentation ROI, integrated over all slices, C1
Intensity_IntegratedIntensity_C1_Corr	Total intensity enclosed by 3D Segmentation ROI, integrated over all slices, C1 after background correction
Intensity_IntegratedIntensity_C1_Enhanced	Total intensity enclosed by 3D Segmentation ROI, integrated over all slices, FIJI Pre-processed C1
Intensity_IntegratedIntensity_C1_Enhanced_Corr	Total intensity enclosed by 3D Segmentation ROI, integrated over all slices, FIJI Pre-processed C1, background corrected
Intensity_IntegratedIntensity_C2	Total intensity enclosed by 3D Segmentation ROI, integrated over all slices, C2
Intensity_IntegratedIntensity_C2_Enhanced	Total intensity enclosed by 3D Segmentation ROI, integrated over all slices, FIJI Pre-processed C2
Intensity_IntegratedIntensity_C2_Enhanced_Corr	Total intensity enclosed by 3D Segmentation ROI, integrated over all slices, FIJI Pre-processed C2, background corrected
Intensity_IntegratedIntensity_C2Corr	Total intensity enclosed by 3D Segmentation ROI, integrated over all slices, C2 after background correction
Intensity_IntegratedIntensity_C3	Total intensity enclosed by 3D Segmentation ROI, integrated over all slices, C3
Intensity_IntegratedIntensity_C3_Enhanced	Total intensity enclosed by 3D Segmentation ROI, integrated over all slices, FIJI Pre-processed C3
Intensity_IntegratedIntensity_C3_Enhanced_Corr	Total intensity enclosed by 3D Segmentation ROI, integrated over all slices, FIJI Pre-processed C3, background corrected
Intensity_IntegratedIntensity_C3Corr	Total intensity enclosed by 3D Segmentation ROI, integrated over all slices, C3 after background correction
Intensity_IntegratedIntensity_C4	Total intensity enclosed by 3D Segmentation ROI, integrated over all slices, C4
Intensity_IntegratedIntensity_C4_Enhanced	Total intensity enclosed by 3D Segmentation ROI, integrated over all slices, FIJI Pre-processed C4
Intensity_IntegratedIntensity_C4Corr	Total intensity enclosed by 3D Segmentation ROI, integrated over all slices, C4 after background correction
Intensity_IntegratedIntensity_C4_Enhanced_Corr	Total intensity enclosed by 3D Segmentation ROI, integrated over all slices, FIJI Pre-processed C4, background corrected
Max_Eccentricity	Maximum eccentricity of any 2D slice of the 3D segmentation
Total_Area	Total area of all 2D segmentations constructing the 3D segmentation
Lifetime	Number of 2D slices that the 3D object is present in

***Note:*** The output from the Python notebook is an Excel file, the same as seen in from the result from step 42. Each Excel file, or equivalent tab in the master file, will have the integrated intensity for each 3D Segmentation Object, which is given by Tracked_Object_Number for each image channel that was input into CellProfiler. The eccentricity of each 2D nuclear segmentation which are used to construct the 3D objects are measured, and the maximum eccentricity measured per set of 2D segmentations constructing a 3D segmentation is given by “Max_Eccentricity.” The number of slices that a 3D segmentation appears in is given by the “Lifetime” measurement. An explanation of all the measurements given in the Excel file can be seen in Table 3.

### Manual validation of the quantification against raw imaging data

**Timing: ∼30 min per embryo**

Following the automated image analysis, the quantified nuclei are validated manually against the unprocessed imaging data, to confirm which nuclei show expression of phospho-SMAD proteins or additional immunofluorescence targets.

50. Normalize the measured immunofluorescence of each nucleus to its DAPI immunofluorescence intensity.
51. Rank the nuclei from high to low, according to their normalized immunofluorescence (Figure 4C).
52. Locate the nuclei with brightest normalized immunofluorescence intensity in the ‘Tracked Object’ output image stack, using the assigned unique identifier.
53. Validate the measure immunofluorescence intensity in the corresponding Z-slice in the unprocessed microscopy file to semi-manually validate (Figure 4).

## Expected outcomes

In Brumm et al.,^1^ we were able to demonstrate that the protocol can be modified to analyses a wide range of sample types: adherent cells, pre-implantation human embryos as well as pre- and post-implantation mouse embryos. Furthermore, the protocol is applicable to an adherent *in vitro* model of mouse embryonic development.^19^ We therefore anticipate that it can be adapted to a range of other pre-implantation mammalian embryos, and cell-based models of development such as blastoids, gastruloids or models of mammalian post-implantation development.

Recent years have seen an increase in the number of models of especially human embryonic development, either by *in vitro* culture of the embryos^20^^,^^21^ or cell-based models, e.g. as reviewed by Shahbazi & Pasque.^22^ This protocol provides the necessary tools to investigate the activity of the TGF-superfamily in these systems, which will shed light on the mechanisms underlying these crucial phases of development.

In pre-implantation human embryos phosphorylated SMAD proteins were first detectable at early blastocyst stage, between 5 and 6 days post fertilization.^1^ Blastomeres without detectable phospho-SMAD proteins may display high, speckled background staining across the entire cell body (Figures 1C and 1D).

When analyzing human blastocysts, the morphology of the embryo may change during acetone treatment. Occasionally, the blastocoel cavity will cave in slightly or collapse entirely. This does not impact the downstream immunofluorescence protocol or image analysis, but may be visible in presented imaging data (Figure 1B).

SHURMS Pipeline segments nuclei in the human blastocysts in 2 spatial dimensions using the StarDist machine learning algorithm in FIJI. The output from FIJI is a set of nuclear segmentation masks. CellProfiler is then utilized to track these 2D nuclear segmentations axially and re-label them as 3D tracked objects (Figure 4A), while measuring the intensity of markers of interest inside each 2D segmentation.

The output from CellProfiler is a csv file with each nuclear segmentation labeled with a corresponding 3D identification number, and a measure of the intensity of each marker of interest inside 2D segmentation mask. Matlab is then used to integrate the tracked 2D nuclear segmentations into 3D nuclear objects using the 3D identification number and integrate the intensity for each marker of interest inside the 3D nuclear objects. The output from Matlab is a spreadsheet with each nuclear object, its volume, and the integrated signal from all the proteins of interest inside that nuclear segmentation. These are separated by embryo.

## Quantification and statistical analysis

The phospho-SMAD immunofluorescence staining displays a high, uniform background staining across the entire cell body in cells lacking phospho-SMAD expression (Figures 1C and 1D), which is especially high in cells located closest to the objective during imaging. It is therefore useful to normalize the phospho-SMAD fluorescence intensity of each tracked nucleus to the corresponding nuclear staining (DAPI fluorescence intensity) to identify nuclei with real phospho-SMAD expression (Figures 4B and 4D). Occasionally, low nuclear staining in apoptotic cells or trophectoderm cells, which are located far away from the objective, will result in artificially high normalized phospho-SMAD immunofluorescence staining (Figure 4D). Therefore, the unique identifier of nuclei with high normalized fluorescence intensity are noted and manually validated in the original microscopy data: locate a nucleus with high DAPI-normalized phospho-SMAD2 fluorescence intensity in the files of tracked nuclei and find the corresponding Z-frame in the original microscopy data to confirm the phospho-SMAD2 expression (Figure 4B).

## Limitations

The primary limitation of the immunohistochemistry protocol is that the rabbit-anti-pSMAD2 antibody (Cell Signaling Technology Cat# 18338) detects phosphorylated SMAD1/5 protein as well.^1^ It is therefore important to investigate whether BMP signaling is active in the biological sample of interest, before interpreting any results obtained from phospho-SMAD2 immunostaining.

Furthermore, both antibodies for detection of phosphorylated SMAD2 and SMAD1/5 (Cell Signaling Technology, Cat# 18338 and Cat# B5B10, respectively) were raised in rabbit and cannot be combined in one analysis. Alternatively, mouse-anti-SMAD2/3 (BD, Cat# 610842) can be used to detect TGFβ/ACTIVIN/NODAL signaling in combination with phospho-SMAD1/5 detection in primed hESCs.^1^

Human embryos are inherently variable, and the quality of immunofluorescence analysis can vary between embryos, even if analyzed in parallel (Figure 1D). Therefore, the quantification of the phospho-SMAD immunofluorescence requires manual validation in the original imaging data. This may not be required for other, more consistent biological materials.

Furthermore, the protocol requires harsh antigen retrieval and permeabilization, which may not be compatible with all protein epitopes. The immunofluorescence detection of other proteins alongside phosphorylate SMAD proteins therefore requires independent optimization.

The image analysis software used is primarily open source, except for Matlab, which will require a license. A corresponding Python code is provided as well, if acquiring a Matlab license is a hardship.

StarDist segmentation will work well on discrete objects in 2D. If the embryo has a great number of overlapping nuclei in 2D where boundaries are indistinct, segmentation and tracking will be suboptimal.

## Troubleshooting

### Problem 1

The phospho-SMAD immunostaining results in high background staining (Step 20).

### Potential solution

Prolonged incubation in warm acetone can contribute to increased background staining. Ensure the acetone and the watch glasses are thoroughly chilled on ice for at least 1 h prior to use in this protocol. Maintain the watch glass on dry ice at all times, unless handling the embryo under the stereo microscope.

Should retrieval of an embryo take longer than 2–3 min, place the watch glass back on dry ice, allow the temperature to drop and then attempt to retrieve the embryo again.

Human embryos are highly variable. Vast differences in immunofluorescence analysis can be observed across different antibodies (Figure 1D).

### Problem 2

The phospho-SMAD and/or additional immunofluorescence staining is dim (Step 20).

### Potential solution

The epitopes of other proteins of interest may be affected by this protocol. Some antibodies may require higher concentration, leave increased background staining, or may lose specificity entirely. Reoptimize any antibodies to be used in combination with phospho-SMAD protein detection, preferably on easily accessible and abundant material.

Permeabilization may have been insufficient. Prepare 1% Triton-PBS and 0.1% Triton-PBS solution fresh on the day and use it to prepare fresh dilutions of methanol.

Visualization of the immunostaining may be challenging. Ensure the microscope settings are adequate. Check the argon laser power is set to 20%. White light laser excitation may be insufficient for detection of phosphorylated SMAD proteins.

### Problem 3

The human embryo is stuck to the watch glass after acetone treatment (Step 12).

### Potential solution

Use a P1000 to squirt a small amount of room-temperature 0.1% Triton-PBS onto the embryo and dislodge it. Quickly retrieve it from the acetone as soon as it can be observed to be moving freely. Do not apply excessive amounts of 0.1% Triton-PBS to the watch glass, as it increases the temperature rapidly. Additionally, the mixing of acetone and PBS causes swirls in the solution, making it harder to retrieve the embryo.

## Resource availability

### Lead contact

Further information and requests for microscopy advice and reagents should be directed to and will be fulfilled by the lead contact, Anna Sophie Brumm (abrumm@ed.ac.uk).

### Technical contact

Further information and requests for computational advice should be directed to and will be fulfilled by the technical contact, Todd Fallesen (todd.fallesen@crick.ac.uk).

### Materials availability

This study did not generate new unique reagents.

### Data and code availability

The image analysis pipeline used to quantify the confocal microscopy data acquired from human blastocyst is made available under: https://doi.org/10.25418/crick.19383428.v1.

The code is also available generalized (i.e. channel labels, not specific markers) at https://doi.org/10.5281/zenodo.15236666.

The accession number for the confocal microscopy data of pre-implantation human embryos, which were analyzed with the presented immunofluorescence detection and image quantification, reported in this paper is EMBL EBI BioImage Archive: S-BIAD1398.

## Acknowledgments

We thank the donors whose contributions have enabled this research. We thank Prof. Dr. Kathy K. Niakan (K.K.N.) for enabling the work on human embryos through funding and facilities. Work in the laboratory of K.K.N. is supported by 10.13039/100010269Wellcome (221856/Z/20/Z) and the Wellcome Human Developmental Biology Initiative (215116/Z/18/Z). Work in the laboratory of K.K.N. is also supported by 10.13039/100010438The Francis Crick Institute, which receives its core funding from 10.13039/501100000289Cancer Research UK (FC001120), the 10.13039/501100000265UK Medical Research Council (FC001120), and 10.13039/100010269Wellcome (FC001120). We thank the laboratory of Dr. Caroline S. Hill for the original immunofluorescence protocol for the detection of phosphorylated SMAD proteins in zebrafish embryos. Crick Advanced Light Microscopy receives its core funding (CC1069) from 10.13039/501100000289Cancer Research UK, the 10.13039/501100000265UK Medical Research Council, and the 10.13039/100010269Wellcome Trust.

## Author contributions

A.S.B. conceived the project. A.S.B. optimized the immunostaining protocol. T.F. and A.S.B. developed the image analysis pipeline. A.S.B. and T.F. wrote the original draft of the manuscript. All authors read and revised the manuscript.

## Declaration of interests

The authors declare no competing interests.
